# Empowering knowledge generation through international data network: the IMeCCHI-DATANETWORK

**DOI:** 10.23889/ijpds.v5i1.1125

**Published:** 2020-02-25

**Authors:** L Otero Varela, M-A Le Pogam, A Metcalfe, PK Kristensen, P Hider, A Patel, H Kim, E Carlini, R Perego, R Gini

**Affiliations:** 1 Department of Community Health Sciences, Cumming School of Medicine, Calgary, Canada; 2 Department of Epidemiology and Health Systems, Center for Primary Care and Public Health, University of Lausanne, Lausanne, Switzerland; 3 Department of Clinical Epidemiology, Aarhus University, Denmark; 4 Department of Population Health, University of Otago, Christchurch, New Zealand; 5 Graduate School of Public Health Dept. of Public Health Sciences; Institute of Aging; and Institute of Health and Environment, Seoul National University, Seoul, Republic of Korea; 6 Istituto di Scienza e Tecnologie dell’Informazione, Pisa, Italy; 7 Agenzia Regionale di Sanità della Toscana, Firenze, Italy

## Abstract

**Introduction:**

The International Methodology Consortium for Coded Health Information (IMeCCHI) is a collaboration of health services researchers who promote methodological advances in coded health information. The IMeCCHI-DATANETWORK initiative focuses on developing a multi-purpose distributed data infrastructure and common data model (CDM) to enable cross-border data sharing and international comparisons.

**Methods:**

IMeCCHI consortium partners from six different countries – Canada, Denmark, Italy, New Zealand, South Korea, and Switzerland – used a questionnaire to describe their original databases which differ in size, structure, content and coding systems. To standardize these data, they agreed on a CDM and mapped their population-based databases to meet the CDM specifications. At the end of this process, local data had a more homogenous content and structure, which made them syntactically and semantically interoperable. Data transformation was performed using a common data management software called *TheMatrix*.

**Results:**

The CDM encompasses four tables of structured data (person characteristics, hospitalizations, outpatient prescription medication and death), linked at the individual level through a person identifier. It can be used to answer research questions across countries using locally converted databases, which facilitates study replication in a distributed fashion. As a proof-of-concept study, an initial research question was addressed using an agreed protocol. Local data were transformed in csv files in the CDM structure and *TheMatrix* was tested to transform the standardized data from each partner into local analytical datasets. This allowed results to be shared between countries, whilst maintaining local control over each region’s data.

**Conclusion:**

The IMeCCHI-DATANETWORK, a model of a distributed data network, demonstrated that it is feasible to analyze international data using standardized analytical methods that enable independent analyses by regions, without relocating datasets thereby protecting local confidentiality obligations. The distributed data infrastructure can produce results that can be generalized to several countries, while facilitating cross-border data sharing and international comparisons.

**Keywords:**

Common data model, international comparison, cross-border data sharing, interoperability, observational data

## Introduction

Over the past decade, numerous health data networks have been developed for comparative effectiveness research, pharmaco- and genetic epidemiology, public health surveillance, and quality or performance assessment [1-4]. Shared data networks have also recently emerged in health services research as a mechanism to understand international variation in health care quality and outcomes [5].

In data networks, each partner has access to routinely collected health data from various sources (e.g., administrative data, electronic health records, population or patient surveys, and/or registry data) [6], and structured and standardized population-based databases are generated using the same program for data cleansing and analysis. Results are comparable by design, thus enabling researchers to answer wide-ranging research questions and make timely decisions at relatively low cost [2,7].

Despite their huge potential, large data networks face important challenges, such as the restraints in data sharing across jurisdictional boundaries due to local regulations about data ownership and security [2,7-9]. Additionally, dissimilar original data features pose a further difficulty for such data networks, as local databases are usually heterogeneous regarding funding (public or proprietary data source), structure (different coding systems) and content [7,10-12]. They combine multiple disparate datasets [2], capturing varied populations, settings and time-periods. Characteristics may vary across time and formal documentation of original data might not be available or up to date [7,9,12]. Furthermore, they have different underlying data models, formats and meanings, which may affect syntactic and semantic interoperability and thereby impact on the quality of the data in the shared data network [9,11,13,14].

To overcome data challenges and enhance study replication, international health data networks have adopted a distributed data infrastructure [9,11,14,15]. Original databases remain under their holder’s control and protection, and are locally transformed to a common data model (CDM) which harmonizes data elements, formats, and sometimes terminologies, while preserving quality [2,14]. The CDM is independent of a specific study requirement [9], but is specific to each data network [16]. Whenever a research question needs to be addressed, a common protocol is developed and embedded in a common data processing script, which is study-specific and purpose-made [2], and is run locally. The outputs of the script are analytical or aggregated datasets that can then be shared to undergo further statistical analyses [2]. So far, several distributed health data networks based on a CDM have been involved in international initiatives demonstrating their ability to generate timely evidence about all aspects of health care [8,11,16,17]. They have also enabled researchers to obtain international insights into limitations including information loss, infeasible data mapping [9, 14], and other data quality issues [13]. However, the distributed networks remain affected by heterogeneity in CDMs, data systems, auditing and search capabilities, as well as local database governance and data privacy regulation [2,13,15]. Some assessment frameworks have thus been proposed to ensure high quality standards for future distributed networks based on CDM [2,10,13,14], including the improvement of transparency in the reporting of data quality measures [18].

In this methodological paper, we therefore aim to report on the development of the IMeCCHI-DATANETWORK multi-purpose distributed data infrastructure and CDM, including its potential strengths and limitations, and address network expansion and research perspectives.

## Methods

### International Methodology Consortium for Coded Health Information (IMeCCHI)

IMeCCHI (https://IMeCCHI.com/) is an international collaboration of health services researchers who promote the methodological advances and applied use of coded health information for disease detection and surveillance, health care quality and patient safety assessment, and more generally, health policy decision-making [19]. 

### Network partners

The IMeCCHI-DATANETWORK initiative currently involves six partners (in Canada, Denmark, Italy, New Zealand, South Korea, and Switzerland), although more collaborators are welcome to join. Current partners are both government and academic employees with a strong interest in working with structured data, although the consortium is now expanding its work to any type of observational health care data including non-coded structured and unstructured data. Spread over four continents, these various countries differ greatly in languages, health care delivery systems, coding terminologies, rules and practices, and also, in the content and structure of routinely collected health databases.

To map their local data holdings into a CDM, each local partner completed a survey to describe their original databases and to provide metadata on the data collection systems from each country. The survey questions focused on: 1) the description and original purposes of each data table, 2) what triggers the creation of a record in each data table and who records the information, 3) data completeness regarding variables/attributes and records, 4) and governance for data access.

### Pilot study and common software

At the same time, a pilot research question was identified, that could be addressed with the data available to the partners. The software chosen to perform common data management was an open source Java-based software called *TheMatrix*, operating on flat csv files using a domain-specific programming language and developed and maintained by the Institute of Science and Information Technology in Italy [2]. The key feature of *TheMatrix* is its ease of use for the data partners: no programming skill is required beyond the ability to extract and transform the original data in csv format, copy-and-paste script files, and execute scripts via a graphical interface. The development of the script is based on a small set of primitive functions and on a programming interface, called *TheMatrixScriptWriter*, that supports the encapsulation of functions for better transparency and for reuse in new scripts.

### Common data model and data mapping

Based on this detailed knowledge of local databases and coding systems, one researcher proposed a draft CDM, based on the results of the survey that accommodated selected variables/attributes from the different original databases that are considered necessary for the future analyses. The partners subsequently modified this draft and reached consensus on a CDM with standardized data format and content.

Local databases were then mapped locally to meet the specifications of the CDM. In other words, each partner extracted the relevant data from their original databases and standardized them according to the CDM specifications. A round of one-on-one calls between the lead researcher and a representative of each data partner established the ETL (extract, transform and load) process of each local database to the CDM. Local coding systems were not mapped to common standardized ones as the CDM contains attributes describing the coding systems – for diagnoses, procedures and drugs – in which the other attributes are coded. They were finally transformed locally using a common script from *TheMatrix*. The CDM tables were stored in comma-separated text files.

At the end of this process, local data had a more homogenous content and structure which made them syntactically and semantically interoperable. Moreover, several research questions and several outcomes can be answered across a number of countries using locally converted databases. So even if the data standardization process is quite long and complex, it remains very efficient as it is performed once, while enabling replicated studies to be undertaken.

## Results

### Mapping Local Databases

The common survey demonstrated that original/local databases were different in size, structure, content and coding systems or terminologies. All the original databases contained different tables linked together through a patient or person identifier. Main tables included data on population characteristics, hospital discharge data, outpatient attendance data, and data from birth and death registries including causes of death (Table 1).

Original databases can be: a) nation-wide databases as in Denmark or New Zealand, b) regional as in Canada or Italy, c) a representative sample of the population as in South Korea, or d) a population subscribing to a healthcare insurance as in Switzerland. Hospital discharge data included a variable number of diagnoses (from 6 to 100) coded in the 9th version of the International Classification of Diseases (ICD9) (one database) and the 10th version (ICD10) (the other five), possibly with local specifications. Procedures were coded in an international standard only in one database (ICD9) and in local coding systems in the others.

Regarding outpatient attendance databases, some databases contained coded diagnostics and procedures and some did not. Coding systems for outpatient prescription drugs were country-specific in all countries, but they all could be mapped to the Anatomical, Therapeutical and Chemical (ATC) system of the World Health Organization (WHO). Drug databases comprised drugs dispensed from community or hospital pharmacies or prescriptions reimbursed by public or private health insurers. All of the countries, expect Switzerland, included information from birth and death registries.

Lastly, some databases contained additional information such as patient satisfaction, congenital anomalies, cancer, traditional medicine treatments, and patient dependency.

### Common Data Model

Based on the results of the survey, a CDM was created, encompassing four tables – person, hospitalizations, outpatient prescription drugs, and death – of structured data linked at the individual level through a person identifier (Table 2).

The person table comprises a few characteristics of the population subjects with gender, dates of birth and death, and dates of entry and exit from the database. The hospitalization table contains: hospital discharge summaries with diagnosis and procedure codes; admission, discharge and procedure dates; and attributes describing local coding systems for diagnoses and procedures. The outpatient prescription drugs table includes information on drug dispensing with dispensing date, local drug code, ATC code, duration of the amount of active principle according to the Defined Daily Dose of the WHO, and an attribute describing the local coding system for outpatient prescription drugs. Finally, the death table contains information on the cause of death.

### Pilot study and data access

The majority of partners (five out of six) declared that they could only access data for a specific study protocol with appropriate governance documents in place. A research question that was deemed to be easy to address with the data and expertise of the partners was care of hip fracture in the elderly. Accordingly, a protocol was developed and submitted to the local governance board for each of the partners. Nearly all consortium partners (four out of five) had the protocol approved and therefore, the next steps were restricted to the data obtained for the pilot study, while the sixth partner applied them to all its database.

### Data transformation and management for the pilot study

The work needed to transform the local data was minimal, and focused on renaming variables, changing date format, or adding empty fields if the fields were missing in the local data. This allowed the data transformation to be performed by the researchers themselves. The exception was South Korea - the mapping proved more difficult for this partner and could not be performed with the available resources.

A data processing procedure to perform the pilot study was developed by one partner using the common software called *TheMatrix*. The open source software was first downloaded and installed by each partner, who then extracted and transformed their local data in csv format compliant with the CDM specifications. A first set of scripts was shared by one of the partners to verify that the datafiles syntactically met the CDM specifications. Execution of a script was controlled via the graphical interface of *TheMatrix*. In a second phase, scripts were developed incorporating the provisions of the pilot study protocol, and they were shared and executed locally. The four partners participating in the study successfully ran the procedure and shared the resulting aggregated tables. 

## Discussion

As an international consortium, IMeCCHI is developing a distributed data infrastructure and CDM across countries. Thus far, it is comprised of six partner countries from four different continents. The CDM encompasses four tables that include information on population characteristics, hospitalizations, outpatient prescription drugs, and death, that is available in all databases and is sufficiently detailed to address a wide range of research questions.

### Comparison with other initiatives of international comparison in health services research 

Compared to other data networks, such as the Organization for Economic Co-operation and Development (OECD) network, the IMeCCHI-DATANETWORK is strengthened by the CDM, which addresses the heterogeneity of data sources across countries, and the need for data correspondents to translate indicator algorithms and codes [20]. In addition, as a distributed research network, IMeCCHI-DATANETWORK makes it possible to share research questions proposed by any of the partners, whereas the OECD network only allows its coordinating agency and expert groups to test new indicators at the international level. Finally, the IMeCCHI-DATANETWORK offers more transparency and replicability in research methods and data processing [21]. Indeed, metric specifications, code lists, classification use over time, linkage methods, risk-adjustment factors, statistical models applied, and the procedure itself are accessible to the research community and the public whereas OECD technical specifications are only accessible to data correspondents or expert groups [22].

### IMeCCHI CDM

As a CDM from a distributed data network, data transformation is performed locally, and thus, original data are never shared, which contributes to overcoming data protection issues and adhering to local privacy laws [2,7,8,9]. Also, characteristics of each local database are described in a common format in a very comprehensive manner, thus making them more homogenous across countries or partners [2,14]. This helps researchers prepare protocols, statistical analyses or interpret comparative results, which are performed in a unique point, limiting misunderstandings or errors.

More specifically to the IMeCCHI CDM, it was designed to keep the data transformation at a minimum, which had two advantages. First, this reduced the time and complexity needed for data transformation to a low level that could be easily managed with the internal resources of the partners in five sites out of six. Second, this avoided information loss, one of the potential pitfalls when mapping original data to a CDM [23]. Indeed, the process of transforming the local information into common semantics is deferred to the moment when a specific research question is addressed. In this way, the local knowledge of the partners is leveraged to build the common variables in a flexible way [10]. For instance, in the pilot study, the interventions after hip fracture were classified into four categories (osteosynthesis, hemiarthroplasty, total hip arthroplasty, and fixation/fusion repair/transfer of the hip joint) and each of the four data partners mapped their local procedure coding system to the four categories. The common procedure could encode the variables, by exploiting the metadata on the coding system, which is recorded by the CDM at the individual level. If necessary, the same technique would allow us to use different coding systems for a partner if they changed over time.

### Limitations posed by the local governance rules

If local partners do not have full access to their data, the local mapping needs to be done on a protocol basis, and this creates a bottleneck for data access. In the IMeCCHI partners this happened in five out of six cases. However, the process of loading the extracted data to the CDM was very fast, and having a single partner developing the data processing procedure ensured that maximal efficiency was attained, within the given constraints. Furthermore, even though the distributed infrastructure grants that data sharing is minimal, data access was not granted to one partner. Unfortunately, it was perceived as being too risky for customers’ privacy by one data owner (i.e. private health insurance). Consequently, the partner was only able to map nationwide hospital discharge data linked over time and hospitals to the CDM. Health insurers are particularly under scrutiny regarding the confidentiality, security and the secondary use of customers’ personal information in the absence of their explicit consent [24], but we hope a wider understanding of the characteristics of distributed networks will be helpful in convincing them that any risk is minimal.

## Conclusion

To conclude, within the IMeCCHI-DATANETWORK initiative, databases from various countries were locally converted into a CDM, allowed study replication in a distributed fashion while granting flexibility in managing data content and maintaining local control over local data. This facilitates international comparisons, and ultimately empowers global knowledge generation on health care services utilization, quality and safety.

## Ethics Statement

This study relied on publically available data and hence was exempt from ethics review and approval.

## Author contributions

LOV drafted the manuscript. MALP, AM, PKK, PH, AP, HK and RG did data mapping and data collection for relative local data holdings, as well as developing the common data model. EC, RP and RG maintained and applied *TheMatrix* software. All authors critically reviewed the paper and approved the final version of the manuscript.

## Supplementary Material

Tables

## Abbreviations

IMeCCHI	International Methodology Consortium for Coded Health Information
CDM	Common Data Model
ICD	International Classification of Diseases
ATC	Anatomical, Therapeutical and Chemical
WHO	World Health Organization
OECD	Organization for Economic Co-operation and Development
