# Supplementary material for: Empowering knowledge generation through international data network: the IMeCCHI-DATANETWORK
Source: Int J Popul Data Sci. 2020 Feb 25;5(1):1125. doi: 10.23889/ijpds.v5i1.1125 (PMC7473294; doi:10.23889/ijpds.v5i1.1125)
Supplement: Tables [file ijpds-05-1125-s001.pdf]

Table 1. Mapping of IMeCCHI-DATANETWORK local databases

COUNTRY	POPULATION DATABASE (N / STARTING YEAR)	HOSPITAL DISCHARGE DATABASE (CODING SYSTEMS)	OUTPATIENT ATTENDANCE DATABASE (CODING SYSTEMS)	OUTPATIENT PRESCRIPTION DRUG DATABASE (CODING SYSTEMS)	BIRTH REGISTRY	DEATH REGISTRY	OTHER DATABASES
**CANADA**	**Alberta vital events statistics for residents** (4.2 million / 1994)	< 2002: **ICD-9-CA / CCP**<br>≥ 2002: **ICD-10-CA / CCI**<br>Up to 25 diagnostic codes<br>Up to 25 procedure codes	**ICD-9-CM**<br>Up to 3 diagnostic codes	Drug dispensed from community pharmacies<br>**ATC codes, Drug Identification Number (DIN)**	Perinatal surveillance system<br>Alberta vital events statistics for residents	Alberta vital events statistics for residents	Patient satisfaction in hospital<br>Congenital anomalies surveillance system<br>Emergency, day-surgery and outpatient clinic data<br>Cancer registry
**DENMARK**	**The patient router file:** persons born, living or working legally Denmark (5.6 million / 2017)	**ICD-10 / NOMESCO**<br>No limit to the number of codes for diagnoses and procedures	No diagnostic code	Reimbursed prescriptions from all community pharmacy<br>**ATC codes**	Medical birth registry	Cause of death registry	Cancer registry
**ITALY**	**Tuscan population assisted by the Regional Healthcare System** (3.6 million / 2004)	**ICD-9-CM**<br>Up to 6 diagnostic codes<br>Up to 6 procedure codes	No diagnostic code	Drug dispensed from community or hospital pharmacies<br>**ATC codes, domestic codes**	Tuscany birth registry	Tuscany death registry	Registry of disease-specific exemptions from co-payment
**KOREA**	**National Health Insurance Database – National Sample Cohort** (NHID-NSC: representative sample cohort of 1 million individuals from South Korea population / 2002)	**ICD-10-KM / EDI**<br>No limit to the number of codes for diagnoses and procedures	**ICD-10-KM / EDI**<br>No limit to the number of codes for diagnoses and procedures	Reimbursed prescriptions from all community or hospital pharmacies in the region<br>**Domestic codes that can be linked to ATC codes**	Birth year data are included in the NHID-NSC	Death year, month and reason data are included in the NHID-NSC	Health examination database
**NEW-ZEALAND**	**Unique national health identifier database** (15 million / 1993)	**ICD-10-AM / ACHI**<br>Up to 99 diagnostic codes<br>Up to 99 procedure codes	No diagnostic code	Medications dispensed by community pharmacies<br>**ATC codes**	National birth registry	National death registry	Cancer registry<br>National Immunization Register<br>National Laboratory Claims Database<br>National Maternity Collection
**SWITZERLAND**	**People aged 65+ insured under the compulsory basic health insurance scheme by one of the biggest health insurance groups** (250,000 / 2010)	≥ 2012: **ICD-10-GM / CHOP**<br>Up to 50 diagnostic codes<br>Up to 100 procedure codes	No diagnostic code (except in one region)	Reimbursed prescriptions from community pharmacies<br>**ATC codes, DIN, domestic codes**	-	-	Measures of dependency for nursing home residents, based on:<br>- Resident Assessment Instrument (RAI);<br>- Computerized planning tool of required nursing (outil de PLAnification Informatisée des Soins Infirmiers Requis, PLAISIR)

Abbreviations: CA, Canadian Modification for diagnosis; CCI/CCP, Canadian Modification for procedures; CM, Clinical Modification; KM, Korean Modification; AM, Australian Modification; GM, German Modification.

Table 2. IMeCCHI-DATANETWORK common data model

TABLE IMECCHI_PERSON

VARIABLES OF THE CDM					Variables of the local data model	
**VARIABLE NAME**	**Description**	**Format**	**Vocabulary**	**Comments and recommendations**	**Table and variable name**	**Comments**
PERSON_ID	Unique person identifier	String		This identifier must be unique across the tables of the same database		
DATE_OF_BIRTH	Date of birth	Date (yyyy-mm-dd)		If the day of the month is unknown, use yyyy-mm-01. If the month is unknown either, use yyyy-01-01		
GENDER	Gender	String	M='male'<br>F='female'<br>O='other'	If gender is unknown, leave missing. If gender changes, create two different time spans (see date_of_entry below) one with the first gender, the second with the other.		
DATE_OF_DEATH	Date of death	Date (yyyy-mm-dd)		If the subject is not dead, leave missing. If day or month of death are unknown, use the same strategy described in the comments to the date_of_birth variable		
DATE_OF_ENTRY	Date when the data tables start to be filled in for this subject	Date (yyyy-mm-dd)		A subject may have several time spans (pairs DATE_OF_ENTRY-DATE_OF_EXIT). DATE_OF_EXIT may be empty if the subject is still registered in the database when the data are extracted.		
DATE_OF_EXIT	Date when the data tables end to be filled in for this subject	Date (yyyy-mm-dd)				

TABLE IMECCHI_HOSP

VARIABLES OF THE CDM					Variables of the local data model	
**VARIABLE NAME**	**Description**	**Format**	**Vocabulary**	**Comments and recommendations**	**Table and variable name**	**Comments**
PERSON_ID	Unique person identifier	String		This identifier must be unique across the tables of the same database		
DATE_OF_ADMISSION	Date of admission	Date (yyyy-mm-dd)				
DATE_OF_DISCHARGE	Date of discharge	Date (yyyy-mm-dd)				
CODE_OF_DIAGNOSIS_1	Code of main diagnosis	String	Coding system for diagnoses adopted in this table by this database			
...						
CODE_OF_DIAGNOSIS_50	Code of the diagnosis number 50	String				
CODE_OF_PROCEDURE_1	Code of procedure number 1	String	Coding system for procedures adopted in this table by this database			
...						
CODE_OF_PROCEDURE_100	Code of procedure number 100	String				
DATE_OF_PROCEDURE_1	Date of procedure number 1	Date (yyyy-mm-dd)				
...						
DATE_OF_PROCEDURE_100	Date of procedure number 100	Date (yyyy-mm-dd)				
CODING_SYSTEM_FOR_DIAGNOSES	A string identifying in which coding system the previous fields of diagnosis are coded	String	String used by each partner, whether it is a standard coding system (for instance, ICD10), or a local modification (e.g. the Canadian modification, coded as "ICD10CAN")	This field will be used to query all the databases with a single rule		
CODING_SYSTEM_FOR_PROCEDURES	A string identifying in which coding system the previous fields of procedures are coded	String		This field will be used to query all the databases with a single rule		

Table IMECCHI_DRUGS

VARIABLES OF THE CDM					Variables of the local data model	
**VARIABLE NAME**	**Description**	**Format**	**Vocabulary**	**Comments and recommendations**	**Table and variable name**	**Comments**
PERSON_ID	Unique person identifier	String		This identifier must be unique across the tables of the same database		
DATE_OF_DISPENSING	Date of dispensing or date of prescription	Date (yyyy-mm-dd)				
CODE_OF_DRUG	Code of the dispensed/ prescribed drug	String	Coding system for drugs adopted in this table by this database			
ATC	ATC of the dispensed/ Prescribed drug	String	WHO table of ATC, to the 5th level			
DURATION	Duration, in days, computed using DDD	Integer		This number is obtained by dividing the amount of active principle in this dispensing/prescription by the DDD, see http://www.whocc.no/ddd/definition_and_general_considera/		
CODING_SYSTEM_FOR_DRUGS	A string identifying in which coding system the previous field of drug is coded	String	String used by each partner, either the standard coding system or a local modification.			
